# Benchtop-fabricated lipid-based electrochemical sensing platform for the detection of membrane disrupting agents

**DOI:** 10.1038/s41598-020-61561-7

**Published:** 2020-03-12

**Authors:** Sokunthearath Saem, Osama Shahid, Adree Khondker, Camila Moran-Hidalgo, Maikel C. Rheinstädter, Jose Moran-Mirabal

**Affiliations:** 1 McMaster University, Department of Chemistry and Chemical Biology, Hamilton, L8S 4L8 Canada; 2 McMaster University, Department of Physics and Astronomy, Hamilton, L8S 4L8 Canada

**Keywords:** Membrane structure and assembly, Chemical physics, Electronics, photonics and device physics, Techniques and instrumentation, Sensors and probes

## Abstract

There are increasing concerns about the danger that water-borne pathogens and pollutants pose to the public. Of particular importance are those that disrupt the plasma membrane, since loss of membrane integrity can lead to cell death. Currently, quantitative assays to detect membrane-disrupting (lytic) agents are done offsite, leading to long turnaround times and high costs, while existing colorimetric point-of-need solutions often sacrifice sensitivity. Thus, portable and highly sensitive solutions are needed to detect lytic agents for health and environmental monitoring. Here, a lipid-based electrochemical sensing platform is introduced to rapidly detect membrane-disrupting agents. The platform combines benchtop fabricated microstructured electrodes (MSEs) with lipid membranes. The sensing mechanism of the lipid-based platform relies on stacked lipid membranes serving as passivating layers that when disrupted generate electrochemical signals proportional to the membrane damage. The MSE topography, membrane casting and annealing conditions were optimized to yield the most reproducible and sensitive devices. We used the sensors to detect membrane-disrupting agents sodium dodecyl sulfate and Polymyxin-B within minutes and with limits of detection in the ppm regime. This study introduces a platform with potential for the integration of complex membranes on MSEs towards the goal of developing Membrane-on-Chip sensing devices.

## Introduction

Bacterial pathogens, pesticides, and parasitic vectors are common water-borne risks that are currently detected and quantified using methods such as ELISA, chromatography, and mass spectrometry. These methods are reliable and offer high precision and accuracy, but are expensive, require highly trained technicians, and are time consuming^1–3^, which precludes their use in resource-limited environments^3–6^. Thus, there is an increasing demand for diagnostic tools that do not compromise affordability, sensitivity, and portability for applications in point-of-care (PoC) diagnostics^3,7^, personalized medicine^8,9^, food quality assessment^10,11^, and water testing^12^. Biosensors are attractive routes to address these needs because they leverage biorecognition elements to offer rapid and low-cost solutions for the detection of potentially harmful agents and can be adapted to portable platforms^13–16^. Particularly relevant to food safety, environmental testing and biosecurity areas is the development of biosensors that can detect the disruption of the cell plasma membrane – a hallmark of the presence of pathogenic microorganisms or toxins that can pose serious threats to human health.

The cell plasma membrane is a complex structure that separates the internal cellular components from external environments. Apart from protecting the cell from its surroundings, the plasma membrane mediates ion and small molecule transport, adhesion, motility, and the uptake of larger foreign bodies through endocytosis. The membrane is primarily composed of a phospholipid bilayer, within which sterols, carbohydrates, and proteins are embedded. These additional components modulate the plasma membrane’s physicochemical properties and biological function. Perturbation of a biological membrane through small molecules and proteins that destabilize the phospholipid bilayer can result in membrane damage, cell lysis, and death. This series of events is of particular concern when the destabilizing agents are of synthetic or pathogenic origin and they result in the lysis of cells. Existing biosensors for the detection of synthetic lytic agents mostly rely on catalytic (e.g., enzyme inhibition) or affinity based systems (e.g., antibodies, aptamers, lectins, and bacteriophages)^13–15,17–20^. The complexity of existing sensing systems presents a unique opportunity for the development of innovative sensing solutions for the detection of lytic agents that are low-cost, portable, rapid, and simple to operate.

The field of biosensors has been rapidly expanding over the past decades, with electrochemical methods as one of the dominant sensing techniques^14,16^. In particular, lipid-based electrochemical biosensors have been proposed as attractive platforms for the rapid and quantitative detection of harmful pathogens. Recent studies used liposomes loaded with a redox mediator to detect hemolytic bacteria^21,22^. In this system, the liposomes burst upon exposure to the lytic agent, which released the redox mediators to produce a quantifiable electrochemical signal. While effective in a laboratory setting, this system requires multi-step kinetics before signal acquisition, a large number of components, extensive sample preparation, and lacks portability and sensitivity. More recently developed lipid-based lytic sensors use supported lipid bilayers (SLBs) or tethered bilayer membranes (tBLMs) on conductive surfaces coupled with electrical impedance spectroscopy (EIS) for the sensing of lytic compounds^23–25^. However, existing systems use planar electrodes and in the case of tBLMs require surface immobilization of a tethering agent which adds additional production time, complexity, and variability to the biosensor. Additionally, most lipid-based sensing systems employ EIS as an electrochemical sensing technique. Although sensitive, EIS can be time consuming due to the use of large frequency sweeps, it is expensive and difficult to miniaturize, and often requires complex data analysis and fitting for quantification^26,27^. The limitations in such lipid-based biosensing technologies for the detection of harmful lytic agents preclude their application for a growing biosensing market.

This work presents a platform that exploits the natural barrier properties of lipid membranes for the electrochemical detection of lytic agents. In this platform, simple and low-cost benchtop microfabrication techniques are used to produce gold microstructured electrodes (MSEs)^28,29^, which are then coated with model membranes composed of 1,2-dimyristoyl-sn-glycero-3-phosphocholine (DMPC). The operating principle of the lipid-based sensor is that while the DMPC membrane is intact, it passivates the MSE shielding it from redox-active molecules in solution and preventing electrochemical signal generation. Exposure to lytic agents permeabilizes the membrane, which reveals the MSE surface to the solution containing redox active reporter molecules and results in the generation of an electrochemical signal proportional to the concentration of the lytic agents. We have performed sensing studies using this platform for the quantitative detection of an ionic surfactant (sodium dodecyl sulfate, SDS) and an antimicrobial drug (Polymyxin-B, PmB). The fabrication of the lipid-based sensors was optimized to produce the fastest response to lytic factors and reduce the material cost. The optimized lipid sensors were able to rapidly (seconds to minutes) and reproducibly detect SDS and PmB, with LODs of 10 and 1 ppm, respectively. The present platform combines affordable bench-top fabrication techniques and electrochemistry to offer a scalable and versatile method that could be used to rapidly detect lytic agents in aqueous samples. The simplicity of the fabrication method suggests that devices incorporating different membrane compositions could be integrated in multiplexed format and at low costs. We anticipate that this type of lipid-based sensor platform could be used in the future within PoC devices where a range of lytic factors can be detected and quantified.

## Results and Discussion

The sensing platform was implemented using model membranes, which simplified the biological membrane down to its major component and allowed us to assess the impact of different device and sample preparation parameters on sensor performance. In the experiments reported we used membranes composed of DMPC, a phospholipid with two 14-carbon aliphatic tails and a zwitterionic phosphocholine head-group, which has been widely used in the preparation of biomimetic membranes^30,31^. The advantages of using DMPC are that it has a transition temperature that is comparable to that of mammalian cell membranes and the interactions of DMPC with components of natural membranes have been studied extensively. DMPC having fully saturated tails also resulted in membranes with high packing density that ensured the best chance of MSE chip passivation. These characteristics provided us with a good starting point for the design of Membrane-on-Chip devices. The main concept behind the sensor platform, discussed in detail below, is that lipids deposited on a structured electrode form a passivating layer that prevents a redox-active reporter in solution from accessing the working electrode surface. This results in negligible current generation during electrochemical sensing with an intact membrane. However, when the DMPC membrane is damaged by a membrane-disrupting agent (*e.g*. surfactants, drugs, pathogen-derived hemolytic peptides) and holes are formed in the passivating layer, the redox reporter molecules can diffuse to the electrode surface and transfer electrons, generating an electrochemical signal. The strength of the signal depends on the composition and concentration of the disrupting agent and the amount of damage caused to the membrane, as well as on the total surface area of the working electrode used in building the sensor. Thus, the platform developed takes advantage of lipid membranes deposited on high surface area structured electrodes and electrochemistry to provide rapid and highly sensitive detection of membrane disrupting compounds.

### Electrode fabrication and characterization

**Figure 1:**
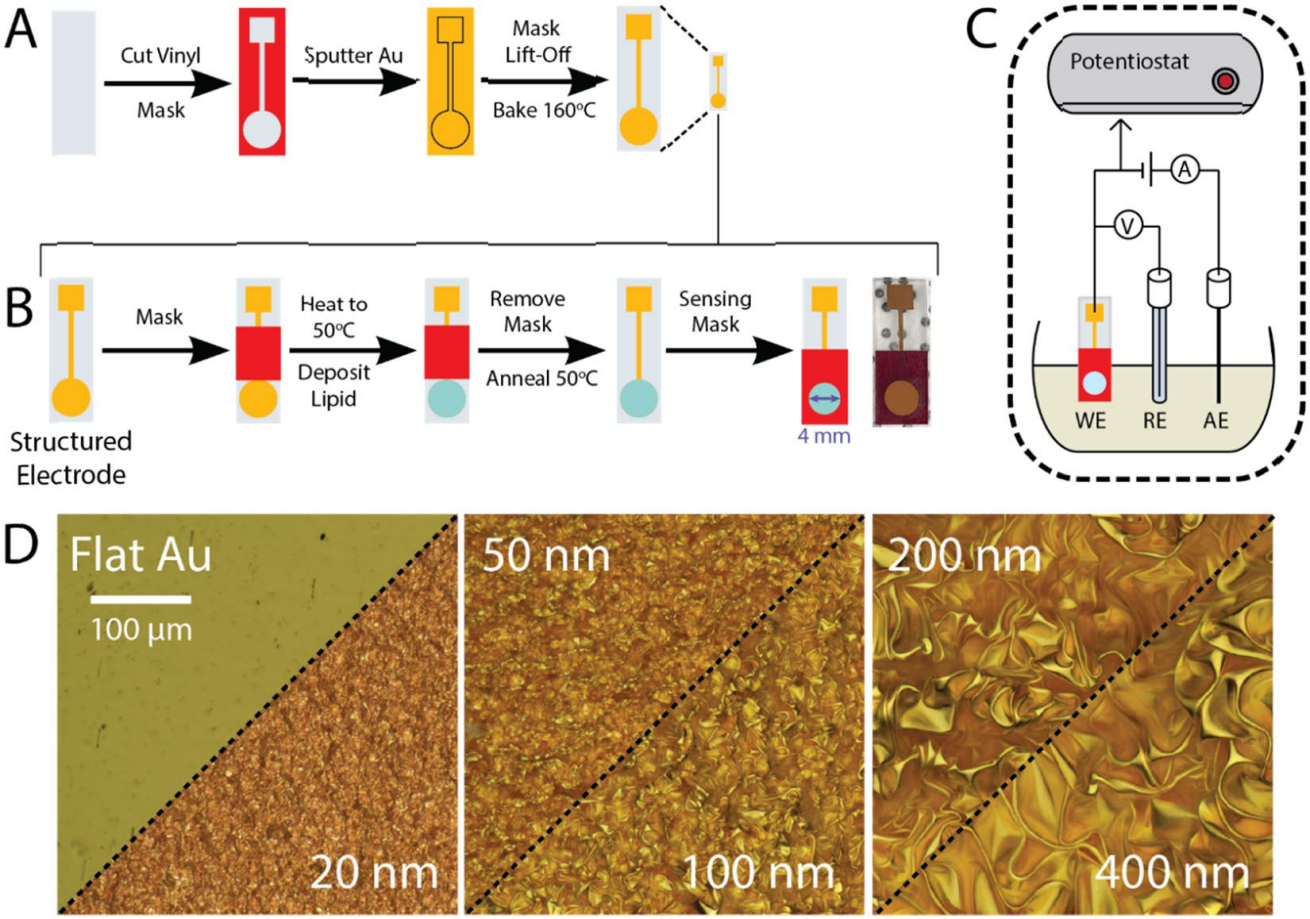
Schematic of the sensor fabrication and operation. (**A**) MSEs were fabricated on pre-stressed polystyrene (clear rectangle), which was masked during gold deposition using an adhesive vinyl stencil (red). After sputtering a thin gold layer (yellow), the stencil was removed to reveal the patterned electrode. Thermal shrinking of the substrate resulted in structuring of the Au film. (**B**) Each MSE was masked to deposit lipid solution only on the sensing pad. The solvent was evaporated, forming a thin lipid film that was annealed under constant temperature and humidity conditions. Prior to sensing, a mask was applied onto the MSE pad to ensure a reproducible membrane sensing surface. (**C**) Schematic of the 3-electrode electrochemical sensing set up. The DMPC-coated MSE was used as the working electrode (WE), with a Pt wire as the auxiliary electrode (AE), and a standard Ag/AgCl reference electrode (RE). (**D**) 3D optical reconstruction of 20-nm-thick flat and 20–400 nm-thick wrinkled Au films. All optical images taken at the same magnification.

MSEs were fabricated using commercial thermo-responsive polymer substrates (pre-stressed PS), as shown in Fig. 1A and previously described^28^. The final transverse dimensions of the shrunken electrodes were 40% of the initial design (*e.g*., the sensing pad diameter was reduced from 12.5 to 5 mm), corresponding to an overall reduction to 16% of the original area. The thermal shrinking of the PS substrate caused the electrode surfaces to buckle, with the resulting wrinkle sizes dictated by the thickness of the gold films. SEM and optical images of the electrode surfaces before and after shrinking (Fig. 1D) show the expected increase in wrinkle size for thicker gold films, which arises from the stress imposed by the shrinking substrate and the mismatch in elastic modulus between the PS and the rigid film^32^. The structured electrodes showed submicron-sized wrinkles for 20-nm-thick Au films and micron-sized wrinkles at Au thicknesses ≥ 50 nm. The wrinkled features were only observed in substrates containing Au films, as images of bare PS substrates before and after shrinking showed that these substrates remained flat. We have previously reported an enhancement of electroactive surface area (ESA) >600% for wrinkled electrodes when compared to flat electrodes with similar footprints^29,32^. Similar enhancements were observed for the electrodes used in this study (Supplementary Information, Fig. S1**)**. The enhanced ESA makes wrinkled electrodes advantageous for use in chip-based electrochemical sensors, since a working electrode with 16% the footprint of a flat electrode will provide similar current output, thus improving device portability and sensitivity. Furthermore, despite the difference in the size of the topographical features, there is no change in the charge transfer efficiency when the thickness of the Au films is increased.

### Lipid-based sensor fabrication and principle of operation

The lipid membrane deposition process is shown in Fig. 1B and explained in detail in the materials and methods section. Briefly, the MSE was masked with a rectangular adhesive vinyl and placed on a 50 °C hotplate for 5 minutes. Fifteen microliters of the DMPC solution were drop cast onto the circular sensing pad. The coated electrodes were then annealed in an oven for 1 hour at 50 °C to form uniform lipid layers. The deposition mask was peeled off and replaced by a sensing mask with a 4 mm-diameter cut-out exposing the sensing pad. Figure 1C shows the 3-electrode electrochemical setup with the DMPC lipid sensor as the working electrode.

**Figure 2:**
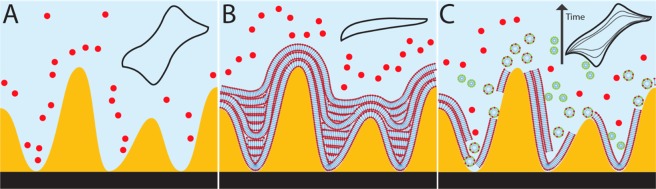
Sensing mechanism of the lipid-based electrochemical sensor. (**A**) Bare MSEs produce the maximum electrochemical signal from a redox reporter in solution (red dots); inset: typical CV curve for KFeCy in solution. (**B**) DMPC membranes deposited on the MSEs passivate the electrode surface, preventing redox processes to occur; inset: CV with no signal. (**C**) When the sensor is exposed to a solution containing membrane-disrupting factors, the membrane degradation exposes the electrode surface and increasing redox signals are measured over time as shown in the inset. Gold surface topography and lipid layer drawings not shown to scale.

The objective of this study was to develop a sensing platform capable of detecting the presence of membrane-disrupting agents in solution by using model DMPC membranes to passivate the surface of gold MSEs. Cyclic voltammetry (CV) was used for the electrochemical detection of membrane damage by factors such as SDS and PmB. The sensing mechanism of the platform (Fig. 2) relies on the electrochemical signal generated by a redox reporter molecule (potassium ferricyanide, KFeCy) added to the solution containing the sample. In CV measurements, when the voltage is cycled between 0 and 0.4 V on a bare MSE, the KFeCy reporter is oxidized and reduced resulting in anodic and cathodic current peaks (Fig. 2A, inset). On the other hand, when a DMPC membrane is present on the MSE sensing pad, the KFeCy cannot access the Au surface to undergo redox processes, thus suppressing the current signal (Fig. 2B, inset). In the presence of a membrane-disrupting factor (Fig. 2C), the DMPC membrane is stripped away until the underlying Au is exposed, leading to the appearance of redox signal from the reporter. The evolution of the current signal is dependent on the concentration of membrane disruptors present in solution. Thus, the performance of the lipid-based electrochemical sensor would be dependent on the maximum electrochemical signal achievable, the time required to reach the maximum signal, and the lowest amount of analyte that could be detected.

### Influence of wrinkle size on sensor performance

The effect of the electrode surface wrinkle size on DMPC membrane conformation and sensing ability was examined by depositing DMPC lipid membranes on electrodes fabricated from 20–400 nm thick films. To ensure full passivation of the electrodes, irrespective of wrinkle size, a DMPC solution with a concentration of 0.5 mg/mL (relatively high for the purpose of this study) was used to prepare the lipid membranes. Prior to testing the sensors against a membrane disrupting agent, each electrode was immersed in a 2 mM KFeCy solution and a 10-segment CV scan was performed, which helped identify current leakage due to unevenly deposited membranes. Once complete passivation was confirmed (*i.e*., no current redox signal formation observed), the DMPC-coated electrode was immersed in a solution containing 2 mM KFeCy and 0.1% SDS, and sensing of the membrane disruption was done via CV. The anionic surfactant SDS was chosen as a test compound because it can disrupt the DMPC membrane through a detergent mechanism **(**Supplementary Information, Fig. S2). The detergent readily disrupts the membrane and the speed of this process is proportional to the concentration of SDS in solution, which makes it an ideal compound for use as a synthetic lytic factor^33^ to test the membrane-based sensors.

**Figure 3:**
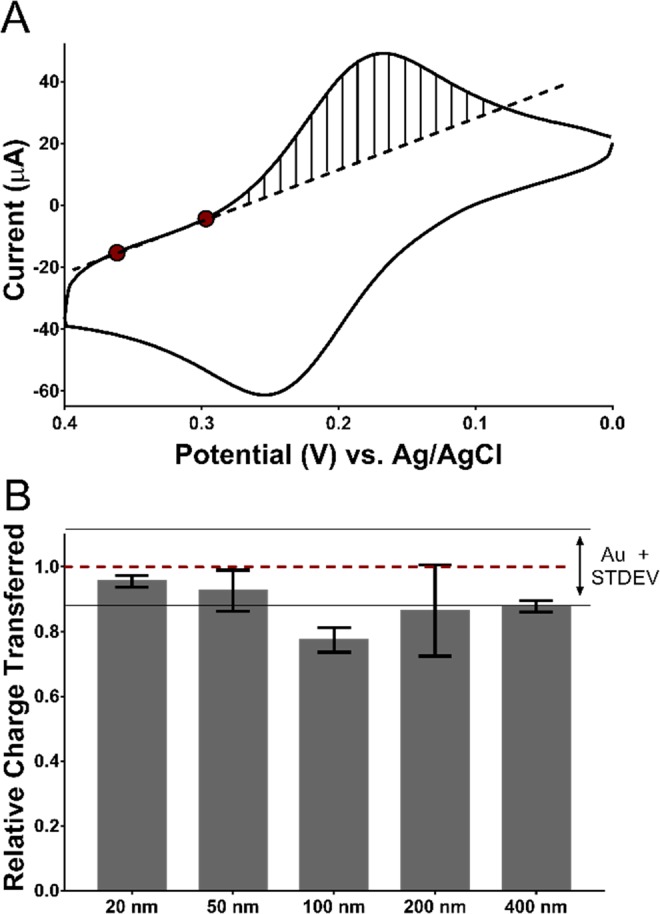
(**A**) Typical cyclic voltammogram of a solution containing 2 mM KFeCy showing the linear regression used for the baseline correction of the cathodic peak. The peak was integrated after the linear regression to obtain the total charge transferred from the redox process. (**B**) Relative charge transfer plot of 20–400 nm MSEs coated with 0.5 mg/mL DMPC after being exposed to a 0.1% SDS solution for 10 minutes. The charge transferred was normalized to bare electrodes without membranes (dotted line). Bars represent the mean values and error bars shown are standard deviations obtained from n = 3 replicate sensors.

Signal arising from membrane disruption was quantified by the integration of the charge transferred during the redox cycling in CV measurements. The membrane-coated sensors were immersed in the SDS-containing solution and sensing was performed at room temperature with no agitation for 10 minutes. As the membrane was disrupted by SDS and more electrode surface became accessible the sweeping voltage in the CV reduced and oxidized the redox reporter (*i.e*., KFeCy) producing cathodic and anodic peak currents that increased over time until reaching a plateau. Quantitative information was obtained from the voltammograms after 10-minute incubation by integrating the cathodic peak current (Fig. 3A), from which the total charge transferred during the reduction process was calculated. To eliminate any capacitive currents generated from non-faradaic processes and avoid integration bias, a baseline correction was established through a linear regression to two points in the reduction sweep, where the current increased linearly with voltage (Fig. 3A). The total charge transferred for each DMPC sensor was normalized to that of its bare Au counterpart (*e.g*., signals from 20 nm MSEs coated with DMPC sensor were normalized to 20 nm MSEs without DMPC), and all experiments were run in triplicate.

A comparison of the relative charge transferred for the DMPC-coated sensors made from gold films of different thicknesses (Fig. 3B) showed that sensors with smaller wrinkles led to higher and more reproducible signals. Full signal recovery (with respect to the bare electrodes) was observed for sensors made with 20 and 50 nm wrinkled surfaces, whereas sensors made with 100, 200, and 400 nm wrinkled surfaces showed slightly lower average signal recovery. Overall, the 20 nm sensors produced the highest signal to variance ratio compared to all other gold thicknesses. The lack of full signal recovery on sensors made from thicker gold films could be attributed to lipid entrapment in the grooves of the wrinkled gold films. The data shown in Fig. 3B suggests that, MSEs with smaller wrinkle spacing and height (i.e. thinner Au films) offer a topography that results in DMPC lipid films that can be more easily removed by a membrane disrupting agent, resulting in a more sensitive membrane-based sensor.

**Figure 4:**
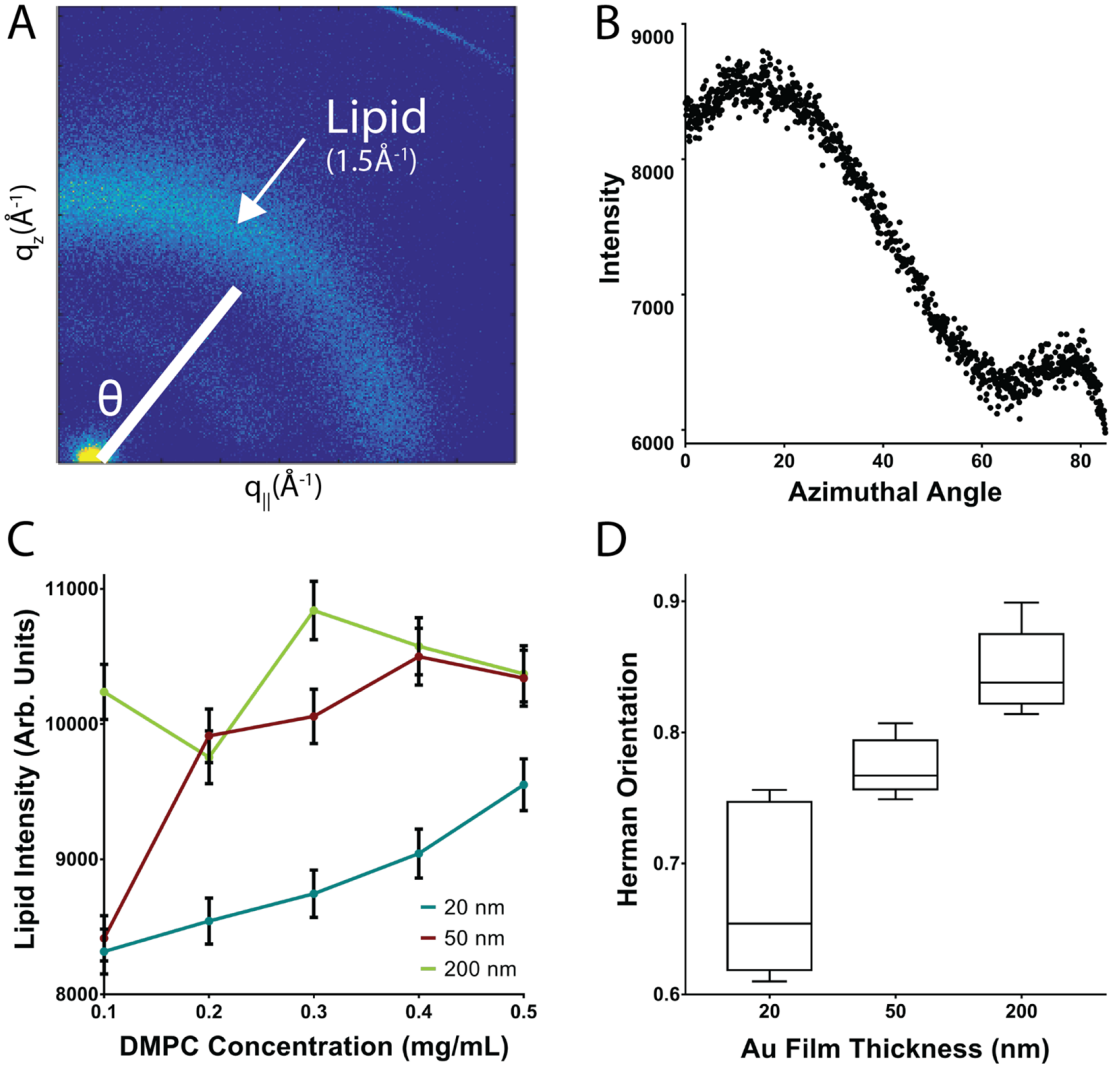
The DMPC membrane conformation on MSEs was assessed through X-ray diffraction. (**A**) A 2D reciprocal-space map of the diffraction intensity obtained from DMPC membranes deposited on MSEs. (**B**) Typical diffracted intensity vs azimuthal angle *Θ* plot. (**C**) Lipid peak intensity signal of DMPC membranes versus different lipid concentrations of the solution deposited. Lines represent data for MSEs made from films with different thicknesses, which led to different wrinkle sizes. (**D**) Plot of Herman’s Orientation parameter versus MSE film thickness.

To further probe the membrane conformation on the MSEs, X-ray scattering experiments were performed on 20, 50 and 200 nm sensors coated with different amounts of DMPC. Figure 4A shows a typical 2D x-ray intensity plot obtained from membranes deposited on an MSE. Analysis of the diffraction data showed no significant signals at *q*-values of *q*_*z*_~0.11 Å^−1^, which would be related to membrane stacking along the out-of-plane (q_z_) axis. The absence of lamellar Bragg peaks, which are intense in thick stacked membrane films^34^, was attributed to the presence of a small number of stacked membranes (<10) along with the high variability in the orientation of the surface topography, which leads to less ordered planar membranes. This would be consistent with the low amounts of DMPC added, which at the lowest concentration would only yield ~10-bilayer-films (if perfectly stacked and uniformly distributed) over the electrode surfaces. The radial pattern of intensity observed at *q*-values of ~1.5 Å^−1^ and plotted in Fig. 4B corresponds to the lipid tail peak (*i.e*., the average distance between two acyl tails in the lipid membranes). This signal is proportional to the total amount of lipids on the area sampled and was therefore used to evaluate the uniformity of lipid deposition on the sensor. Figure 4C shows the total lipid content measured for MSEs coated with increasing lipid amounts. It can be observed that 20 and 50 nm sensors show trends of increasing intensity as more lipids were added, while the 200 nm sensor shows random behavior. Furthermore, the observation that 20 nm MSEs exhibited a linear trend with respect to the concentration of the lipid added implies that casting DMPC on the surface of these electrodes results in a more uniformly distributed membrane film. The radial integration of the lipid peak signal contained within the azimuthal angles 10° < θ < 60° also allowed us to determine the degree of orientation of the membranes by using Herman’s orientation (HO) function:

$$H=\frac{3}{2} < {\cos }^{2}\theta  > -\frac{1}{2}$$

where a value of *H* = 1 represents a perfectly oriented bilayer, whereas a value of *H* = 0.25 represents a membrane with randomly oriented lipids. Typical values for highly oriented lamellar lipid bilayers on flat silicon substrates have been reported in the range of *H* = 0.95–0.98^35^. The HO values for membranes deposited on 20, 50, and 200 nm MSEs increased with increasing wrinkle size (0.65, 0.77 and 0.84 for 20, 50 and 200 nm respectively, Fig. 4D). The lowest HO value, corresponding to the least order and/or higher membrane curvature was observed for 20 nm MSE. This suggests that the DMPC membranes formed on the surfaces with smaller wrinkles conformed better to the topography, which coupled to a more uniform coverage of the electrodes lead to more randomly oriented lipid bilayers. These results together with the electrochemical measurements indicate that sensors made using 20 and 50 nm MSEs provide the most uniformly deposited membranes with the highest signal to variance in the signal resulting from exposure to high concentrations of membrane disrupting factors. Thus, 20 and 50 nm MSEs were used for all subsequent optimization and sensing experiments.

### Optimization of membrane sensor response

The temporal response of the lipid-based sensing platform depends on the ability of the redox reporter to reach the electrode surface after the passivating membrane is damaged. Therefore, we sought to optimize the membrane deposition parameters to obtain the thinnest possible membranes while preserving complete passivation prior to exposure to the disrupting agents. The rationale behind this approach was that a thinner DMPC membrane would require a lower concentration of disrupting factors and less time for them to strip away the lipid membranes, allowing a faster and more sensitive electrochemical response. First, the ability to fully passivate 20 and 50 nm-thick MSEs was tested by depositing the membranes from solutions with increasing lipid concentrations (0.05–0.5 mg/mL). To ensure that the fabricated DMPC films were uniform, we started by using previously reported conditions for the deposition of lipids from solution^36,37^. Briefly, the DMPC solution was drop cast onto the MSE and the sensor was placed in a vacuum chamber for 24 hours followed by a 24 hours annealing at 100% RH and 50 °C. These lipid membrane deposition conditions were used as a starting point to optimize the passivation of the MSEs for sensing applications.

**Figure 5:**
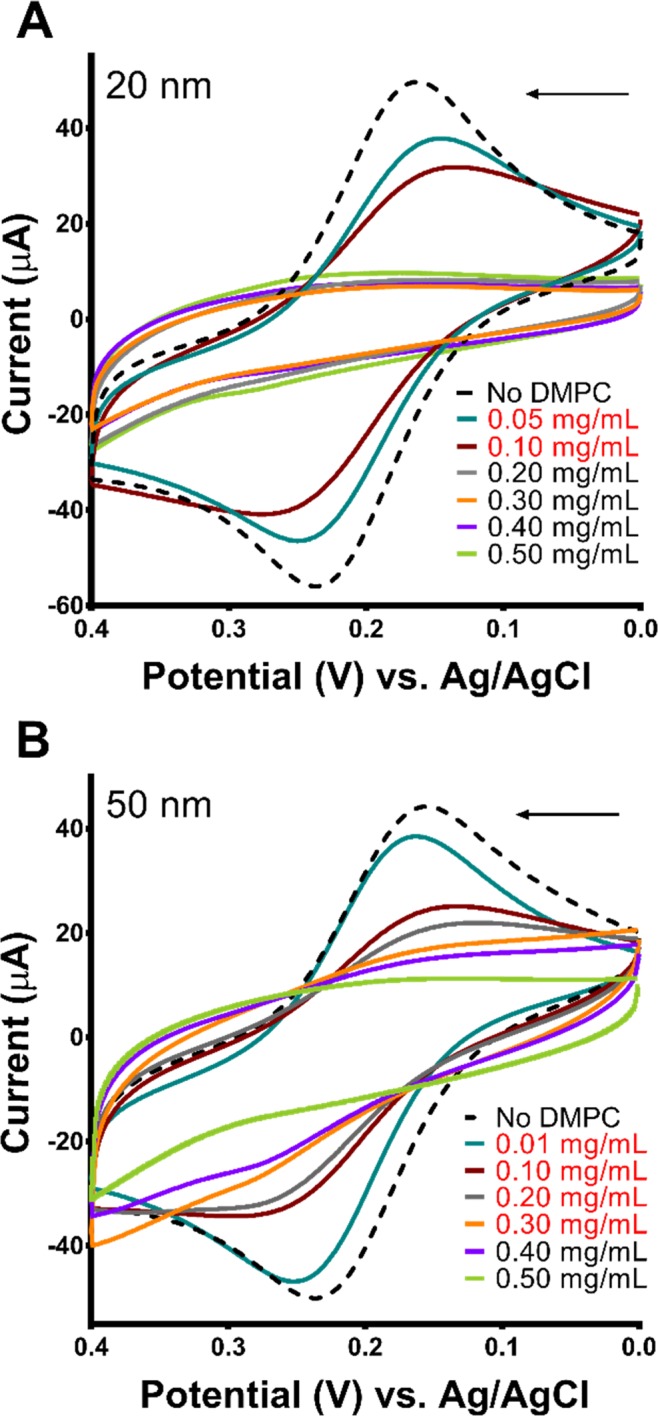
Cyclic voltammograms compare the current signals obtained as the concentration of DMPC used to passivate (**A**) 20 nm and (**B**) 50 nm MSEs was increased. All CV scans were performed in 2 mM KFeCy sensing solution in PBS. Concentrations of DMPC highlighted in red indicate incomplete passivation.

After membrane deposition and annealing, the current leakage was assessed by sensing through CV in solutions containing 2 mM KFeCy, in the absence of any disrupting agents. Figure 5 shows that at low lipid concentrations, the electrode surfaces were not fully passivated and allowed the generation of current peaks (leakage current). As the lipid concentration in the casting solution was increased, the surface was progressively less accessible and eventually became completely passivated. Full passivation was reached at a lipid concentration of 0.2 mg/mL for 20 nm MSEs, while a concentration of 0.4 mg/mL was required for 50 nm electrodes. The observation that electrodes with smaller wrinkles can be passivated by a smaller amount of DMPC can be explained by the topography, where smaller grooves would be easier to fill with lipids and result in a more even coating during drying and annealing. On the other hand, as the wrinkles become larger (*i.e*., on MSEs made from thicker gold films) membranes have to cover topographies with deeper grooves, which leads to the non-uniform deposition of lipids and the formation of aggregates entrapped within the grooves until enough material is deposited to coat the tips of the wrinkles. These results are also consistent with our X-ray diffraction observations that electrodes with smaller wrinkles aid in the production of more uniform DMPC membranes. In view of the membrane deposition and uniformity results, we opted to use 20 nm MSEs for subsequent optimization and sensing experiments. An additional advantage of using thinner gold films for the fabrication of the membrane-based sensors was the reduction of the materials cost per sensor, which decreased from $0.20 for 50 nm-thick films to $0.08 for 20 nm-thick ones.

The DMPC membrane preparation was further optimized by studying the effect of temperature, relative humidity, vacuum, and annealing time – variables that have been reported to affect membrane integrity. All membrane sensors for this optimization were prepared using 20 nm MSEs and 0.2 mg/mL DMPC solution and were immersed in a 0.1% SDS aqueous solution containing 2 mM KFeCy for 10 minutes to determine the membrane stability using CV. In these experiments, the goal was to obtain the most stable membranes (*i.e*., lowest relative charge transfer value for each condition tested), which would translate into sensors with higher reproducibility. No change in membrane stability was observed (Supplementary Information, Fig. S3) when the sensors were prepared: i) using solvent extraction in a vacuum for different lengths of time (0, 1, 4, and 24 hours), while maintaining humidity (100% RH), temperature (50 °C), and annealing time (24 hours) constant; ii) annealing under different humidity conditions (ambient, 98% and 100% RH), while maintaining the annealing time (24 hours), annealing temperature (50 °C), and vacuum time (24 hours) constant; or iii) using different annealing times (0.5, 1, 6 and 24 hours), maintaining humidity (100% RH), temperature (50 °C), and vacuum time (24 hours) constant. The only factor that impacted membrane stability was the annealing temperature, where sensors prepared at 50 °C produced the lowest charge transfer response at 1 minute of exposure to SDS when compared to those annealed at 25, 30, and 40 °C (Supplementary Information, Fig. S3), indicating a more stable membrane. While 50 °C resulted in a slower response, it still yielded full signal recovery (as compared to a bare electrode) at 10 minutes and, more importantly, presented the most reproducible passivation, with fewer sensors showing current leakage. Thus, the sensor preparation conditions were fixed for all subsequent experiments as no-vacuum solvent extraction and 1 hour annealing at 50 °C and ambient RH, which yielded reproducible stable DMPC membranes.

The stability of the DMPC membrane formed on the MSEs was further tested by performing 20 rinse and dry cycles. The membrane sensors were rinsed with water and dried under a nitrogen stream followed by characterization by cyclic voltammetry in sensing solution. The overlaid cyclic voltammograms (Supplementary Information, Fig. S4A) showed no leakage current even after 20 rinse and dry cycles indicating stable DMPC membrane passivation of the MSEs. In addition, we tested the membrane stability in relevant background sensing solutions that would typically be used in practical clinical applications such as serum and blood in addition to 1xPBS that we have used for sensing experiments. The results (Supplementary Information, Fig. S4B) showed no DMPC membrane damage when performing electrochemical sensing in 1xPBS, 10% FBS in DMEM, and human red blood cells even after 10-minute incubations. We also evaluated the possibility of reusing the electrodes after the deposition of a DMPC membrane, sensing and washing (Supplementary Information, Fig. S5**)**. We observed that we could recover the full the electroactive surface area of the MSE after a washing step using soap and isopropyl alcohol. SEM imaging of the MSE surfaces before and after sensing and washing revealed no changes or damage to the gold surface upon DMPC removal (Supplementary Information, Fig. S6**)**. The combined results from electrochemical and surface characterization support the ability to reuse the devices, which contributes to reduce the total cost of the sensing platform.

To evaluate the advantage of using MSEs in the lipid-based sensor platform, we tested planar Au electrodes passivated with DMPC membranes under conditions identical to those used with MSE devices (Supplementary Information, Fig. S7). We monitored the signal generation for planar electrode devices and MSE devices in a 1000 ppm SDS solution containing 2 mM KFeCy. Bare 20 nm Au MSEs produced ~27% higher charge transferred signal compared to planar Au electrodes. Once DMPC membranes were used to passivate the electrodes, planar electrodes produced ~30% signal recovery and MSEs produced ~89% signal recovery compared to their respective control signals with no membrane passivation. These two effects combined translate into a 3.8-fold increase in signal recorded from lipid-based MSE sensors over lipid-based planar electrode sensors. Thus, the enhancement in the total charge transferred and signal recovery make MSEs an ideal choice to produce lipid-based electrochemical biosensors.

### Electrochemical sensing of membrane disrupting factors

To demonstrate the quantitative capabilities of the lipid-based sensor platform, sensing experiments were performed against two molecules known to degrade lipid membranes through a detergent-like mechanism, an anionic surfactant (SDS) and a cationic antibiotic (PmB). SDS was used as a model disrupting factor because its mechanism of action is well understood. Similarly, PmB is a cyclic cationic polypeptide antibiotic produced by the soil bacterium *Paenibacillus polymyxa*, which is capable of dissolving the hydrophobic regions of lipid membranes^38^.

**Figure 6:**
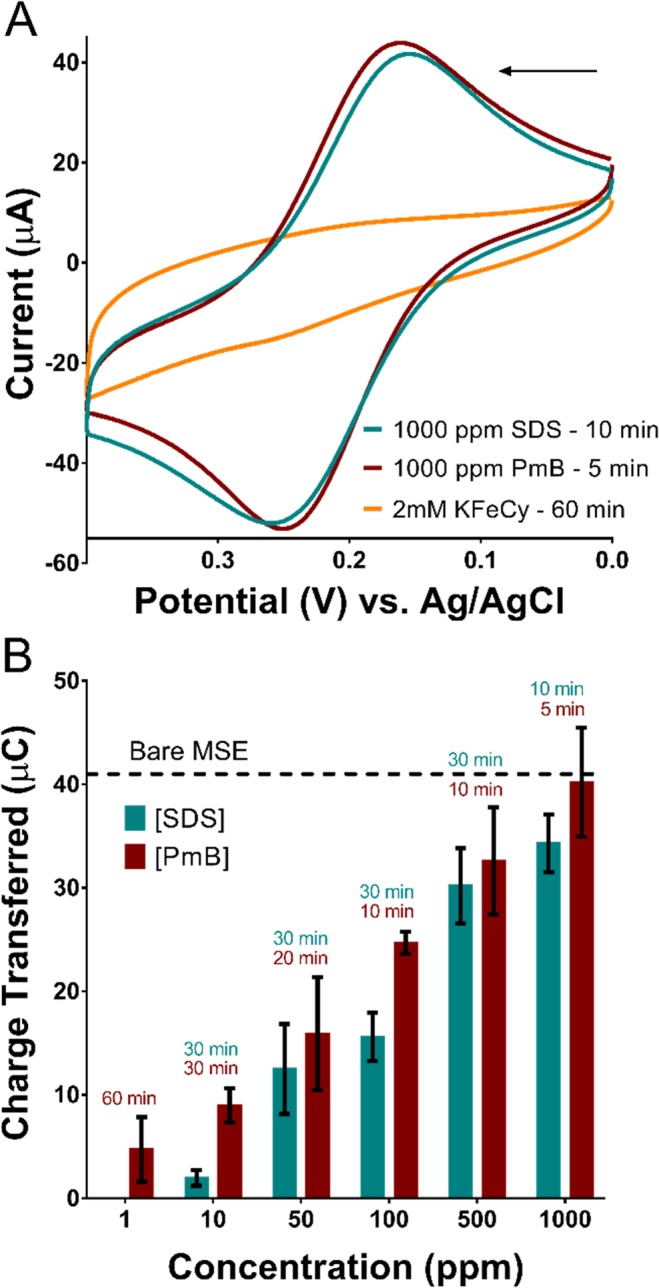
Electrochemical sensing of SDS and PmB at concentrations from 1 to 1000 ppm in presence of 2 mM KFeCy redox reporter. (**A**) Cyclic voltammogram overlaying sensing response to KFeCy (in yellow), SDS (in cyan), and PmB (in red). (**B**) Total charge transferred from sensors incubated with solutions containing increasing concentrations of SDS or PmB compared to charge transferred from bare MSEs (black dashed line). Times required for the sensor to reach the peak electrochemical signal are noted above each bar. Reported charge transfer values are means and error bars are standard deviations of n = 3 replicates.

SDS and PmB were effective in disrupting the membrane on the lipid-based sensor, with high concentrations producing the maximum signal possible from the redox reporter, indicating the complete removal of the membrane. Figure 6A shows typical cyclic voltammograms obtained from sensing experiments performed in sample solutions containing the highest concentrations tested for SDS and PmB. Sensors tested in solutions containing 1000 ppm SDS (cyan curve) or PmB (red) showed appreciable electrochemical signals that increased over time and reached the peak signal after 10 and 5 minutes, respectively. This shows that the membrane was actively disrupted, allowing the redox reporter to access the MSE surface, until a point where the maximum membrane removal possible was reached, leading to a levelling off of the signal. On the other hand, sensors immersed in only 2 mM KFeCy solutions (yellow) did not generate any appreciable redox signal even after 60 minutes immersion in the solution. This was expected, since the synthetic DMPC membrane passivating the electrodes should not be dissolved or ruptured in the presence of the redox reporter molecule alone. Further sensing experiments were performed in solutions containing SDS or PmB at concentrations ranging from 1 to 1000 ppm. In all cases, the electrodes were left in the test solutions until the redox signal reached the peak intensity, and the total charge transferred in the cathodic peak was quantified (Fig. 6B). The time for the sensors to reach the peak signal varied with concentration and is reported above each bar in the graph. As anticipated, SDS and PmB sensing experiments showed an increase in signal generated as the concentrations of the membrane disruptors increased. In particular, sensors incubated with PmB at a concentration of 1000 ppm showed signals comparable to those generated by bare MSEs (dotted line), showing complete removal of the passivating membrane. The sensors reliably detected SDS (*i.e*., produced integrable peaks in the CV curves) down to a concentration of 10 ppm, while PmB was reliably detected at concentrations down to 1 ppm. Since the sensors did not yield integrable electrochemical signals below these concentrations, the limit of detection for SDS and PmB was established as 10 and 1 ppm, respectively.

The DMPC membrane-based sensors produced higher signals and faster responses for PmB solutions than for SDS. This increased PmB response can be attributed to the difference in the structure and the membrane disruption mechanism between SDS and PmB. Both molecules rely on electrostatic interactions with the DMPC head group to initiate membrane disruption. In the case of SDS, the binding event initiates the insertion of the hydrophobic tail into the lipid bilayer, which destabilizes and displaces DMPC molecules from the membrane. The insertion and replacement of lipids by SDS also causes an increase in the membrane fluidity. When enough lipids are displaced, the membrane loses its structural integrity resulting in pore formation and ultimately in the complete removal of the membrane (Supplementary Information, Fig. S2). The membrane disruption mechanism of PmB, on the other hand, has been described using the carpet and the insertion Barrel-Stave models^39^. Initially, PmB peptides interact electrostatically with the DMPC head groups in the membrane, orienting themselves parallel to its surface and forming aggregates^40^. This leads to thinning of the lipid membrane and compromised lipid bilayer integrity resulting water uptake. This process happens rapidly at high PmB to lipid concentrations, which explains the rapid response time for electrochemical sensing of PmB at 100 to 1000 ppm (Fig. 6B). The PmB peptides then insert themselves into the membrane to form pores as described by the Barrel-Stave model. Pore formation causes increased membrane instability and increases the water uptake until the membrane is completely disrupted^41^. Furthermore, the head group of PmB consists of 10 amino acids, which makes it much larger than that of SDS, meaning that each PmB molecule can displace more DMPC than SDS and PmB causes greater membrane destabilization than SDS. Thus, the structural properties and mechanism for membrane destabilization explain why a faster response and lower limit of detection are observed for PmB *vs*. SDS. In view of the detection capabilities of this lipid-based platform, we propose that these sensors could provide advantages in sensitivity, turnaround time, affordability, and portability over current assays for the detection of membrane disrupting agents.

## Conclusion

In this study, we developed an inexpensive electrochemical biosensor platform for the sensitive and rapid detection of membrane disrupting agents by combining benchtop fabricated MSEs with phospholipid membranes. DMPC membranes were used to passivate the structured electrodes, preventing the interaction of a redox reporter with the electrode surface while the membrane remained intact. When the sensors were incubated with solutions containing a membrane-disrupting agent (*i.e*., SDS, PmB), pores formed within the membranes that allowed the reporter molecules to reach the electrode surface and undergo redox cycling, generating an electrochemical signal. We evaluated relevant conditions in the sensor fabrication protocol (*i.e*., gold wrinkle size, membrane deposition and annealing conditions) and determined that the deposition of DMPC membranes from 0.2 mg/mL solutions onto MSEs made from 20 nm-thick Au films, followed by annealing at 50 °C for 1 hour produced the most reproducible and sensitive biosensors. Furthermore, the stability of the membrane sensors to rinsing and drying cycles and to incubation in complex matrices (i.e., serum and whole blood) was demonstrated. Using the optimized conditions for biosensor fabrication, SDS and PmB were detected within minutes down to concentrations of 10 and 1 ppm, respectively, highlighting the excellent sensitivity afforded by the MSEs. This lipid-based electrochemical biosensing platform offers fast detection times, low fabrication costs, great stability to handling and sensing in complex matrices, reproducibility, and represents a first step towards the realization of Membrane-on-Chip devices with potential for future applications in diagnostics targeted against infectious bacteria, field testing for harmful pesticides, and antimicrobial drug testing.

## Methods

### Structured electrode fabrication

All electrodes were fabricated on pre-stressed polystyrene (PS) films (Graphix Shrink Film, Graphix, Maple Heights, OH, USA). PS films were washed with isopropanol (IPA), ethanol (EtOH), and 18.2 MΩ cm water baths (5 minutes each) under orbital agitation (60 rpm) and dried under a nitrogen stream. Adhesive vinyl sheets (FDC-4300, FDC graphic films, South Bend, IN, USA) were cut into stencils to define the electrode shapes using a Robo Pro CE5000–40-CRP blade cutter (Graphtec America Inc., Irvine, CA, USA) equipped with a CB09UA super-steel blade. The vinyl stencils (Fig. 1A) were peeled and transferred onto the clean shrink films, which were cut with the blade cutter into individual rectangular substrates. The PS substrates were then placed in a Torr Compact Research Coater CRC-600 manual planar magnetron sputtering system (New Windsor, NY, USA), and 99.999% purity gold (LTS Chemical Inc., Chestnut Ridge, NY, USA) was sputtered onto the PS substrates at a rate of 0.5 Å/s to the desired thickness. The argon plasma was generated using a 70 mA DC current and a gas flow of 5 sccm. After Au deposition, the vinyl stencils were peeled-off from the substrates, revealing patterned gold electrodes. The electrodes (PS + Au film) were placed in an oven at 160 °C for 5 minutes to thermally shrink the PS substrate and structure the electrodes. The microstructured electrodes (MSEs) were stored in a sealed container until further needed.

### Lipid deposition

Immediately before lipid deposition, the MSEs were rinsed with IPA, EtOH, 18.2 MΩ cm water and dried under a dry nitrogen stream. The electrode surfaces were further cleaned through exposure to UV/O_3_ (185 nm and 254 nm, PSD-UV Benchtop UV-Ozone Cleaner, Novascan, IA, USA) for 15 minutes at room temperature, followed by a 5-minute air plasma treatment (30 sccm air flow, 600 mTorr) in a PDC expanded oxygen plasma cleaner (Harrick, Ithaca, NY, USA) operated in high power setting (30 W). DMPC (1,2-dimyristoyl-sn-glycero-3-phosphocholine, Avanti Polar Lipids Inc, Alabaster, AL, USA) solutions (0.05–0.5 mg/mL) were made using a 1:1 mixture of chloroform and 2,2,2-trifluoroethanol (CHCl_3_:TFE) as a solvent. The electrodes were masked with a rectangular vinyl adhesive mask that left only the circular sensing pad area exposed (Fig. 1B). Once masked, the electrodes were placed on a hot plate preheated to 50 °C for 5 minutes. Then, 15 μL of the DMPC solution were deposited dropwise onto the circular electrode sensing pad. The electrodes were left on the hotplate for 5 minutes to ensure complete solvent evaporation. The effect of vacuum, relative humidity, annealing temperature and annealing time were studied to optimize the DMPC lipid bilayer formation on the MSEs. Following DMPC deposition, the lipid-MSEs were annealed at atmospheric pressure or in vacuum, at temperatures between 25–50 °C, for various lengths of time, and at various relative humidity conditions. The results of these optimization tests are summarized in Supplementary Information, Fig. S3. Briefly, we determined that vacuum, humidity, and annealing duration had no significant contribution to the desired electrode passivation effect. However, annealing at 50 °C showed approximately 30% greater MSE passivation compared to devices made at room temperature. Therefore, the optimal annealing conditions of 50 °C for 1 hour were used. After the annealing process, the electrodes were left to cool in a desiccator for 10 minutes, and the drop casting mask was removed and replaced by a sensing mask (Fig. 1B). The electrodes were then used for electrochemical sensing with the sensing mask in place.

### DMPC stability study

To determine DMPC membrane stability against common matrix solutions like 1xPSB, fetal bovine serum (FBS), and human blood (human red blood cells – HRBC), the DMPC-MSEs were submerged in the respective medium for 10 minutes and sensed using cyclic voltammetry in a 2 mM potassium ferrocyanide solution. Human blood was generously donated to MCR by the Canadian Blood Services (SAGM RBC LR, Ottawa, Ontario, CA). FBS was prepared to a 10% concentration in Dulbecco’s Modified Eagle Medium (DMEM).

### Electrochemical sensing

Potassium ferricyanide (KFeCy – K_4_[Fe(CN)_6_]·3H_2_O, Sigma-Aldrich, Oakville, ON, Canada) was used as redox-active reporter to generate an electrochemical signal when performing cyclic voltammetry (CV) measurements. All sensing solutions used in this study consisted of 2 mM KFeCy in 1x PBS (137 mM NaCl, 2.7 mM KCl, 8 mM Na_2_HPO_4_ and 2 mM KH_2_PO_4_, pH = 7.4, Sigma-Aldrich, Oakville, ON, Canada). For initial membrane disruption experiments, a variable amount of sodium dodecyl sulfate (SDS, Sigma-Aldrich, Oakville, ON, Canada) was added to the sensing solution to achieve the desired SDS concentration. CV measurements were performed using a CHI600E electrochemical workstation (CH Instruments, Austin, TX, USA) in a three-electrode electrochemical cell setup, where the structured electrode was used as the working electrode (WE), a platinum wire was used as auxiliary electrode (AE), and an Ag/AgCl electrode served as the reference electrode (RE). During sensing, all three electrodes were connected to the electrochemical work station and submerged in 15 mL of the sensing solution. The CV experiments were performed with a voltage sweep from 0 to 0.4 V at 0.1 V/s scan rate for 10 segments. A sensing solution with Polymyxin B (PmB) (Sigma-Aldrich, Oakville, ON, Canada) was prepared and tested for membrane disruption in the same way as SDS solutions. To completely remove leftover lipids after sensing and recycle the electrodes, they were washed successively with IPA, EtOH, and 18.2 MΩ cm water, and dried with a dry nitrogen stream. The clean electrodes were stored in a desiccator to be reused.

### X-ray scattering

X-ray scattering data was obtained using the Biological Large Angle Diffraction Experiment (BLADE) at McMaster University. BLADE uses a 9 kW (45 kV, 200 mA) CuKα rotating anode at a wavelength of 1.5418 Å. Both source and detector are mounted on movable arms, such that the sample stays horizontal during measurements. The beam was focused using multi-layer optics through a 200 mm collimator resulting in a high intensity beam with monochromatic X-ray intensities up to 10^8^ counts/s and detected on a HyPix300 two-dimensional detector. The sensors were scanned at a temperature of T = 28 °C and ~50% relative humidity (RH). The two-dimensional intensity maps were then used for quantification of lipid organization within the sample. A powder diffraction peak was observed ~1.5 Å^−1^, corresponding to lipid-lipid distances within a lamellar phase. The spacing in real-space may be determined from d = 2π/q_T_ from Bragg’s Law. The peak intensity, proportional to the presence of organized lipids upon the sample surface, was determined from radially integrating from 1.4 < q_z,||_ < 1.6 for each sample.

## Supplementary Information

Supplementary Information.
