# Supplementary material for: Benchtop-fabricated lipid-based electrochemical sensing platform for the detection of membrane disrupting agents
Source: Sci Rep. 2020 Mar 12;10:4595. doi: 10.1038/s41598-020-61561-7 (PMC7067837; doi:10.1038/s41598-020-61561-7)
Supplement: Supplementary file 1 — Supplementary Information. [file 41598_2020_61561_MOESM1_ESM.pdf]

Supporting Information: Benchtop-fabricated lipid-based electrochemical sensing platform for the detection of membrane disrupting agents

Sokunthearath Saem,[1] Osama Shashid,[1] Adree Khondker,[2] Camila Moran-Hidalgo,[1] Maikel C. Rheinstädter,[2] and Jose Moran-Mirabal[1,*]

[1]McMaster University, Department of Chemistry and Chemical Biology, Hamilton, L8S 4L8, Canada
[2] McMaster University, Department of Physics and Astronomy, Hamilton, L8S 4L8, Canada
[*]mirabj@mcmaster.ca

Supplemental Materials and Methods

Optical microscopy

White light, bright field electrode images were taken with a Nikon Eclipse LV100ND advanced research-grade upright microscope optical microscope (Nikon, Mississauga, ON, CA) fitted with a 50x TU Plan Fluor air objective (N.A. 0.8, W.D. 1.0 mm), and a Nikon DS-Ri2 Camera with a resolution of 4908×3264 pixel and a pixel-size of 7.3×7.3 µm. The images were 3D reconstructed using NIS-Elements Documents software at a z-step size of 1 µm.

DMPC stability study

To determine DMPC membrane stability against common matrix solutions like 1xPSB, fetal bovine serum (FBS), and human blood (human red blood cells – HRBC), the DMPC-MSEs were submerged in the respective medium for 10 minutes and sensed using cyclic voltammetry in a 2 mM potassium ferrocyanide solution. Human blood was generously donated from the Canadian Blood Services (SAGM RBC LR, Ottawa, Ontario, CA). FBS was prepared to a 10% concentration in Dulbecco's Modified Eagle Medium (DMEM).

Supplemental Figures

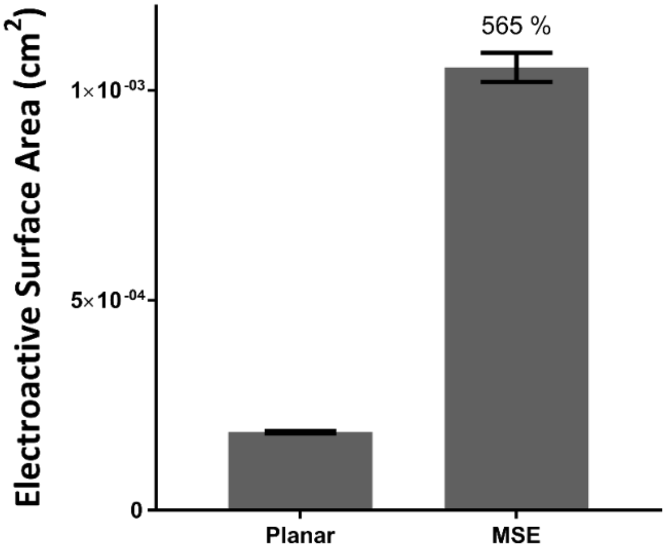

Figure S1. ESA comparison between planar Au electrode and MSEs. The ESA was obtained using CV scanning in a 50 mM H_2SO_4 solution. The MSEs contains improved ESA compared to the planar electrodes allowing for increased sensitivity in electrochemical detection.

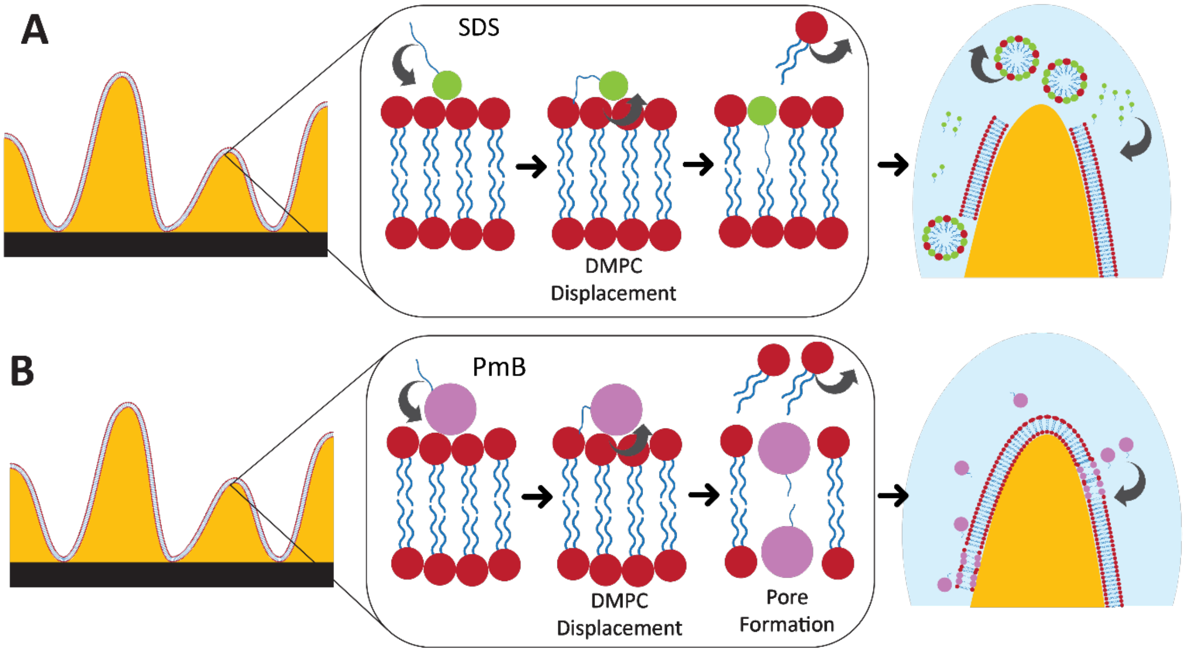

Figure S2. Schematic representation of (a) SDS and (b) PmB membrane disruption mechanisms.

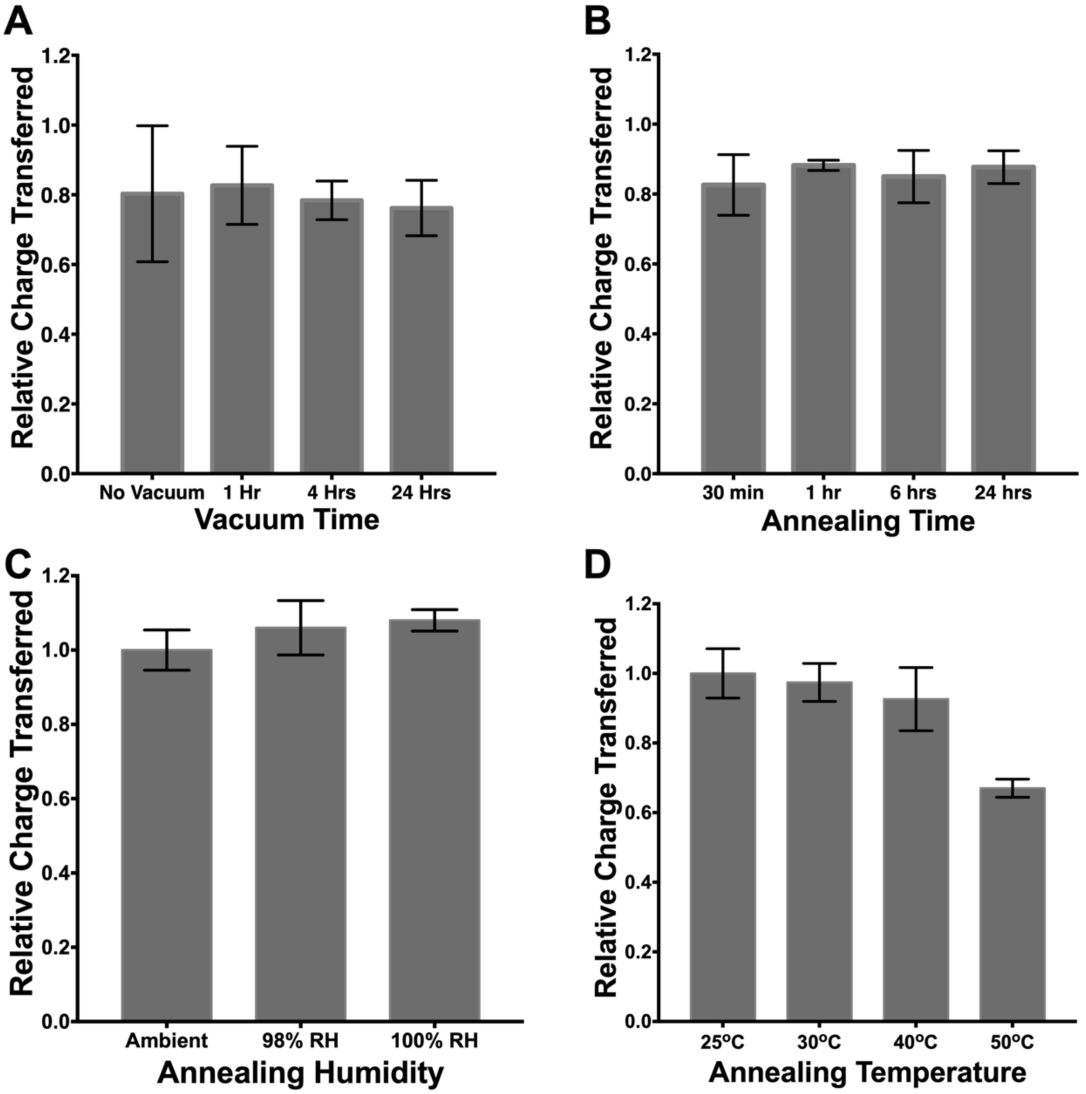

Figure S3. Optimization of vacuum, annealing time, relative humidity and annealing temperature for lipid membrane deposition. DMPC films were made on 20 nm MSEs by drop casting 15 µL from a 0.5 mg/mL stock lipid solution. In all cases sensing was performed through CV in 0.1% SDS solutions in presence of 2mM KFeCy redox reporter. All charge transfers are relative to bare Au sensors with no DMPC. (**a**) Relative charge transferred for sensors prepared using solvent evaporation in vacuum for various lengths of time, while keeping 100% RH and 24-hour annealing at 50°C. (**b**) Relative charge transferred for sensors prepared using solvent evaporation in vacuum for 24 hours, and annealing at 100% RH and 50°C over a variable length of time. Trials were also performed on non-annealed electrodes, but signal leakage was observed prior to exposure to SDS. (**c**) Relative charge transferred for sensors prepared using solvent evaporation in vacuum for 24 hours, and annealing at variable humidity for 24 hours at 50°C. (**d**) Relative charge transferred for sensors prepared using solvent evaporation in vacuum for 24 hours, and 100% RH and 24-hour annealing at variable temperatures.

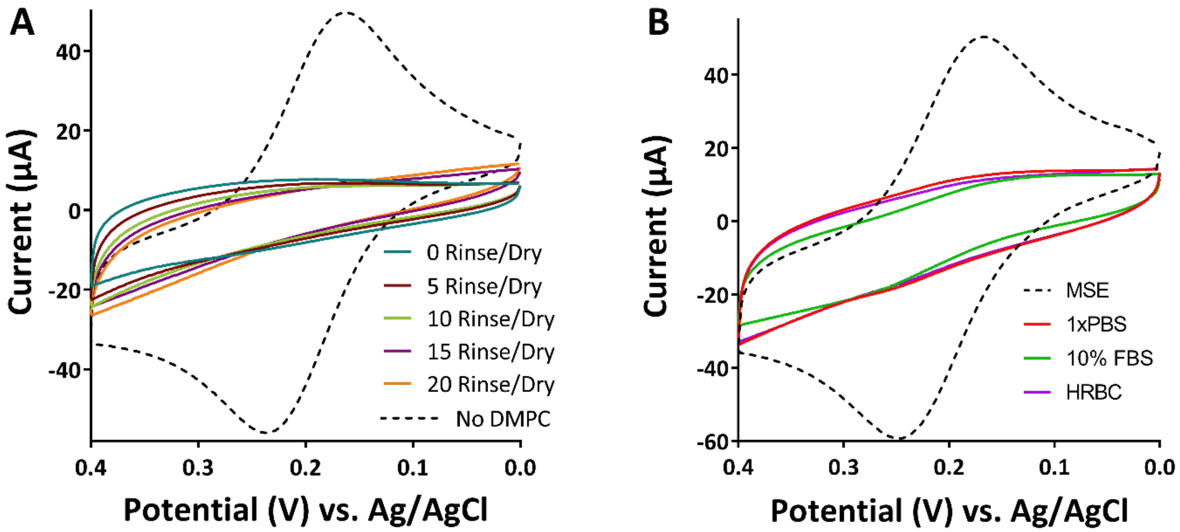

Figure S4. Overlaid cyclic voltammograms showcasing (a) DMPC membrane stability on MSEs through a series of rinsing and drying cycles with water, where the lack of signal demonstrates the membranes were not damaged. (b) DMPC membrane stability in different sensing matrices including 1xPBS, 10% fetal bovine serum (FBS) in DMEM, and human blood (indicated as human red blood cells – HRBC).

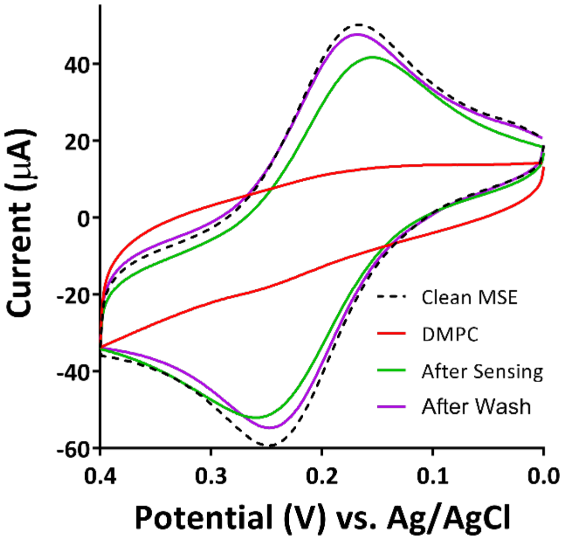

Figure S5. Overlaid cyclic voltammograms showing full device functionality and electroactive surface area recoverability after membrane passivation, sensing, and washing of the MSEs.

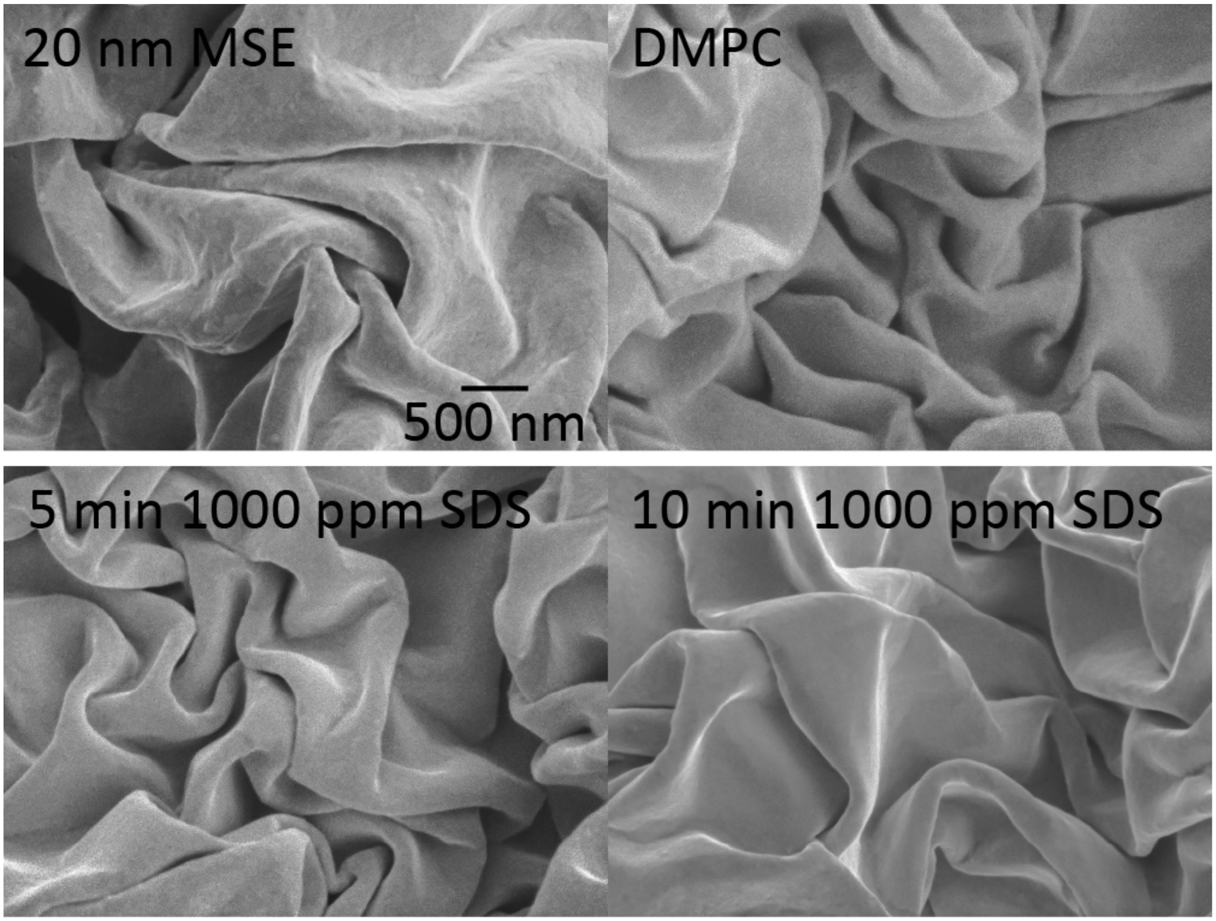

Figure S6. Scanning electron images of 20 nm gold MSEs without DMPC, passivated with DMPC, 5 minutes exposure to 1000 ppm SDS, and 10 minutes exposure to 1000 ppm. The images show no damages to the MSEs upon use and DMPC removal. No noticeable topographical change was observed between the electrode conditions. This further suggests that the MSEs can be reused post analysis without affecting the device functionality.

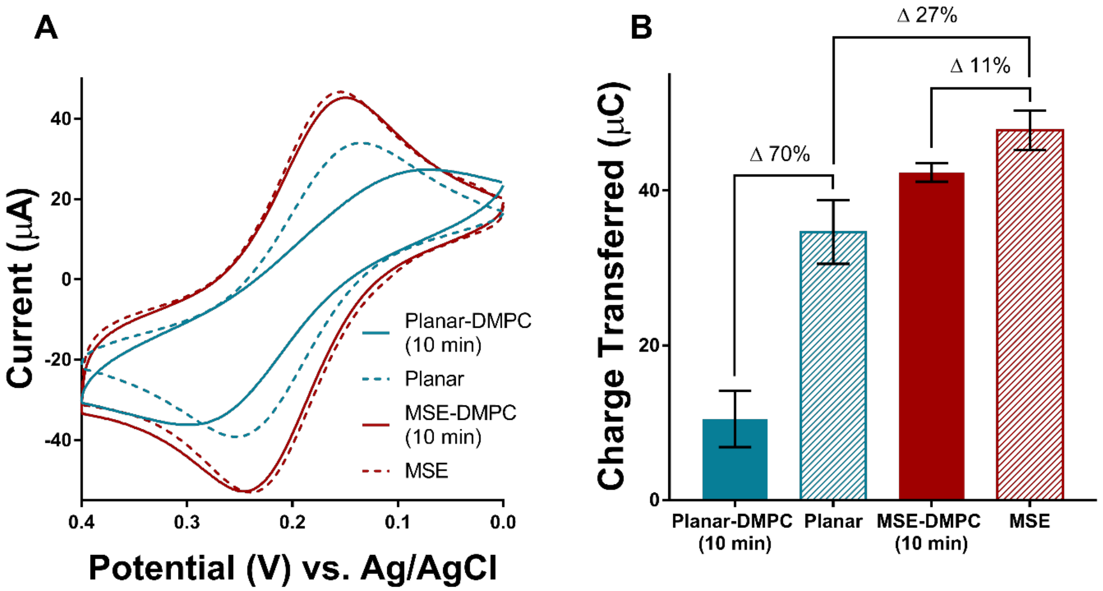

Figure S7. Comparison of the signal generated from 20 nm Au planar electrodes (teal) and MSE (red) passivated with 0.2 mg/mL DMPC membrane. Cyclic voltammetry was performed in a 1000 ppm SDS and 2 mM KFeCy solution until the maximum membrane removal was achieved at 10 min. (a) MSE-DMPC devices showed the maximum signal at 10 min comparable to bare MSEs (dotted line). On the other hand, planar Au electrodes did not reach the maximum signal. (b) Quantification of CV plots in charge transfer showing signal close to maximum signal generation from MSE-DMPC devices while planar-DMPC devices only regenerated approximately 30% signal. Furthermore, bare MSEs (striped red bar) showed approximately 27% higher signal output over bare planar Au electrodes (striped teal bar), making MSEs the favourable option for high sensitivity electrochemical sensing applications.
